# *In vivo* protection against ZIKV infection and pathogenesis through passive antibody transfer and active immunisation with a prMEnv DNA vaccine

**DOI:** 10.1038/npjvaccines.2016.21

**Published:** 2016-11-10

**Authors:** Karuppiah Muthumani, Bryan D Griffin, Sangya Agarwal, Sagar B Kudchodkar, Emma L Reuschel, Hyeree Choi, Kimberly A Kraynyak, Elizabeth K Duperret, Amelia Anne Keaton, Christopher Chung, Yinho K Kim, Stephanie A Booth, Trina Racine, Jian Yan, Matthew P Morrow, Jingjing Jiang, Brian Lee, Stephanie Ramos, Kate E Broderick, Charles C Reed, Amir S Khan, Laurent Humeau, Kenneth E Ugen, Young K Park, Joel N Maslow, Niranjan Y Sardesai, J Joseph Kim, Gary P Kobinger, David B Weiner

**Affiliations:** 1 The Wistar Institute, 3601 Spruce Street, Philadelphia, PA, USA; 2 Special Pathogens Program, National Microbiology Laboratory, Public Health Agency of Canada, Winnipeg, Manitoba, Canada R3E 3R2; 3 Department of Medical Microbiology and Infectious Diseases, University of Manitoba, Winnipeg, Manitoba, Canada R3E 0J9; 4 Inovio Pharmaceuticals Inc., Plymouth Meeting, PA, USA; 5 Division of Infectious Diseases, The Children's Hospital of Philadelphia, Philadelphia, PA, USA; 6 Department of Molecular Medicine, University of South Florida Morsani College of Medicine, Tampa, FL, USA; 7 GeneOne Life Science Inc., Teheran-Ro, Gangnam-Gu, Seoul, Korea

## Abstract

Significant concerns have been raised owing to the rapid global spread of infection and disease caused by the mosquito-borne Zika virus (ZIKV). Recent studies suggest that ZIKV can also be transmitted sexually, further increasing the exposure risk for this virus. Associated with this spread is a dramatic increase in cases of microcephaly and additional congenital abnormalities in infants of ZIKV-infected mothers, as well as a rise in the occurrence of Guillain Barre’ syndrome in infected adults. Importantly, there are no licensed therapies or vaccines against ZIKV infection. In this study, we generate and evaluate the *in vivo* efficacy of a novel, synthetic, DNA vaccine targeting the pre-membrane+envelope proteins (prME) of ZIKV. Following initial *in vitro* development and evaluation studies of the plasmid construct, mice and non-human primates were immunised with this prME DNA-based immunogen through electroporation-mediated enhanced DNA delivery. Vaccinated animals were found to generate antigen-specific cellular and humoral immunity and neutralisation activity. In mice lacking receptors for interferon (IFN)-α/β (designated IFNAR^−/−^) immunisation with this DNA vaccine induced, following *in vivo* viral challenge, 100% protection against infection-associated weight loss or death in addition to preventing viral pathology in brain tissue. In addition, passive transfer of non-human primate anti-ZIKV immune serum protected IFNAR^−/−^ mice against subsequent viral challenge. This study in NHP and in a pathogenic mouse model supports the importance of immune responses targeting prME in ZIKV infection and suggests that additional research on this vaccine approach may have relevance for ZIKV control and disease prevention in humans.

## Introduction

Zika virus (ZIKV) is a single-stranded, positive sense RNA flavivirus,^1^ spread primarily through the bite of infected *Aedes* mosquitos.^2–4^ However, during the recent outbreak in South and Central America, novel mechanisms of ZIKV transmission have been described including sexual and transplacental transmission.^5–7^ The virus is endemic in parts of Africa and Asia and has spread unabated through South America, Mexico and the Caribbean over the last 2 years.^8,9^ Factors including increased global travel and an expansion of the range of *Aedes* mosquitos owing to climate change portend further spread of this virus, expanding its range in the southern United States over the next few years.^5,9^

ZIKV infection presents with a prodrome of myalgias, arthralgias, malaise and low-grade fever with a rash appearing approximately 7 days post infection that may occur with conjunctivitis and retro-orbital pain. The clinical presentation is similar to, albeit less severe than, chikungunya and dengue viral infections, which are also transmitted through the same mosquito vectors. During the French Polynesian outbreak in 2013, an increased risk of Guillain Barre’ syndrome was identified in infected individuals.^4,6,10^ Alarmingly, during the recent outbreak in South and Central America, microcephaly and other congenital abnormalities in infants have been observed in mothers who were infected by ZIKV during pregnancy.^11–13^ In April 2016, the United States Centers for Disease Control and Prevention confirmed the link between ZIKV infection and microcephaly establishing ZIKV as a teratogen. There are currently no licensed therapies or vaccines against ZIKV infection. Therefore, the development and evaluation of potential vaccines to control and halt the spread of this rapidly emerging infectious agent is of high priority.^14^ Here we describe the development and evaluation of a synthetic ZIKV prME DNA vaccine delivered by electroporation for its immunogenicity and its impact on ZIKV infection in a pathogenic animal challenge model.

## Results

### Construction of the ZIKV-prME consensus DNA vaccine

**Figure 1:**
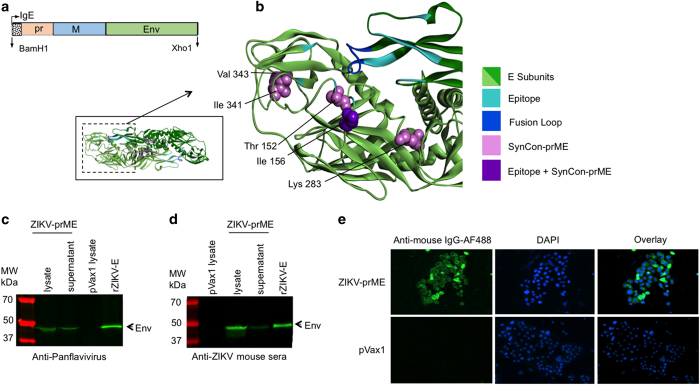
Construction of the ZIKV-prME consensus DNA vaccine. (**a**) Diagrammatic representation of the ZIKV-prME DNA vaccine indicating the cloning of prME into the pVax1 mammalian expression vector. A consensus design strategy was adopted for the ZIKV-prME consensus sequence. Codon-optimised synthetic genes of the prME construct included a synthetic IgE leader sequence. The optimised gene construct was inserted into the BamH1 and Xho1 sites of a modified pVax1 vector under the control of the CMV promoter. (**b**) Model building of the ZIKV-E proteins demonstrates overlap of the vaccine target with potentially relevant epitope regions. Several changes made for vaccine design purpose are located in domains II and III (located within dashed lines of inset, middle left). Vaccine-specific residue changes in these regions are shown in violet CPK format on a ribbon backbone representation of an E Env protein dimer (each chain in light and dark green, respectively). Regions corresponding to the defined EDE are indicated in cyan, and the fusion loop is indicated in blue. Residue Ile156 (T156I) of the vaccine E protein, modelled as exposed on the surface of the 150 loop, is part of an N-linked glycosylation motif NXS/T in several other ZIKV strains as well as in multiple dengue virus strains. (**c** and **d**) Expression analysis by SDS-PAGE of ZIKV-prME protein expression in 293T cells using western blot analysis. The 293T cells were transfected with the ZIKV-prME plasmid and the cell lysates and supernatants were analysed for expression of the vaccine construct with pan-flavivirus (**c**) or Sera from ZIKV-prME immunized mice (**d**). Protein molecular weight markers (kDa); cell lysate and supernatant from ZIKV-prME transfected cells and rZIKV-E positive control were loaded as indicated. (**e**) Immunofluorescence assay (IFA) analysis for ZIKV-prME protein expression in 293T cells. The cells were transfected with 5 μg of the ZIKV-prME plasmid. Twenty-four hours post transfection, immunofluorescence labelling was performed with the addition of sera (1:100) from ZIKV-prME immunised mice followed by the addition of the secondary anti-mouse IgG-AF488 antibody for detection. Staining with sera from ZIKV-prME and pVax1 immunised mice is shown. DAPI panels show control staining of cell nuclei. Overlay panels are combinations of anti-mouse IgG-AF488 and DAPI staining patterns. DAPI, 4',6-diamidino-2-phenylindole; ZIKV-prME, precursor membrane and Env of Zika virus.

A consensus sequence of ZIKV prM (precursor membrane and Envelope (Env) genes (ZIKV-prME) was generated using prM and Env sequences from various ZIKV isolated between the years of 1952 and 2015, which caused infection in humans. The ZIKV-prME consensus sequence was cloned into the pVax1 vector after additional modifications and optimisations were made to improve its *in vivo* expression including the addition of a highly efficient immunoglobulin E (IgE) leader peptide sequence (Figure 1a). Optimal alignment of ZIKV-Env sequences was performed using homology models and visualisation on Discovery Studio 4.5. Reference models included PDB 5JHM and PDB 5IZ7. Aligned residues corresponding to specific regions on the prME antigen were labelled in the models for visualisation purposes (Figure 1b). The optimised consensus vaccine selections are, in general, conservative or semi-conservative relative to multiple ZIKV strains analysed in this study. Structural studies of EDE-specific neutralising antibodies have revealed that these recognition determinants can be found at a serotype-invariant site at the Env–dimer interface, which includes the exposed main chain of the fusion loop and two conserved glycan chains (N67- and N153-linked glycans).^15^ These two glycosylation sites are not highly conserved in other flaviviruses. Moreover, ZIKV does not possess the N67-linked glycosylation site, and the N154-linked glycosylation site (equivalent to the N153-linked glycosylation site in dengue) is absent in some of the isolated ZIKV strains. As part of the consensus design, we therefore designed the construct leaving out this glycosylation site. Lack of glycosylation at this site has been correlated with improved binding of EDE1 type broadly neutralising antibodies (bnAbs) to ZIKV-Env protein.^15^

Subsequent to construction, expression of the ZIKV-prME protein from the plasmid was confirmed by western blot analysis and an indirect immunofluorescence assay (IFA). The protein extracts prepared from the cells transiently transfected with ZIKV-prME were analysed for expression by western blot using a panflavivirus antibody (Figure 1c) and sera collected from ZIKV-prME immunised mice (Figure 1d). ZIKV-prME expression was further detected by IFA by the staining of 293T cells transfected with ZIKV-prME plasmid at 48 h post transfection with anti-ZIKV-prME specific antibodies (Figure 1e).

### ZIKV-prME DNA vaccine induces antigen-specific T cells in C57BL/6 mice

**Figure 2:**
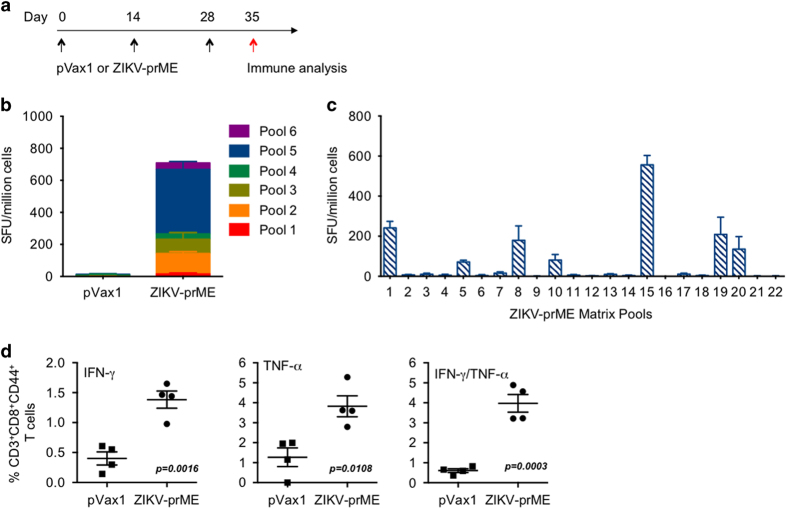
Characterisation of cellular immune responses in mice following vaccination with the ZIKV-prME DNA vaccine. (**a**) Timeline of vaccine immunisations and immune analysis used in the study. (**b**) ELISpot analysis measuring IFN-γ secretion in splenocytes in response to ZIKV-prME immunisation. C57BL/6 mice (*n*=4/group) were immunised i.m. three times with 25 μg of either pVax1 or the ZIKV-prME DNA vaccine followed by electroporation. IFN-γ generation, as an indication of induction of cellular immune responses, was measured by an IFN-γ ELISpot assay. The splenocytes harvested 1 week after the third immunisation were incubated in the presence of one of the six peptide pools spanning the entire prM and Env proteins. Results are shown in stacked bar graphs. The data represent the average numbers of SFU (spot-forming units) per million splenocytes with values representing the mean responses in each±s.e.m. (**c**) Epitope composition of the ZIKV-prME-specific IFN-γ response as determined by stimulation with matrix peptide pools 1 week after the third immunisation. The values represent mean responses in each group±s.e.m. The experiments were performed independently at least three times with similar results. (**d**) Flow cytometric analysis of T-cell responses. Immunisation with ZIKV-prME induces higher number of IFN-γ and TNF-α secreting cells when stimulated by ZIKV peptides. One week after the last immunisation with the ZIKV-prME vaccine, splenocytes were cultured in the presence of pooled ZIKV peptides (5 μM) or R10 only. Frequencies of ZIKV peptide-specific IFN-γ and TNF-α secreting cells were measured by flow cytometry. Single function gates were set based on negative control (unstimulated) samples and were placed consistently across samples. The percentage of the total CD8^+^ T-cell responses are shown. These data are representative of two independent immunisation experiments. IFN, interferon; TNF, tumour necrosis factor; ZIKV-prME, precursor membrane and Env of Zika virus.

The ability of the ZIKV-prME plasmid vaccine to induce cellular immune responses was evaluated. Groups of four female C57BL/6 mice were immunised with either the control plasmid backbone (pVax1) or the ZIKV-prME plasmid vaccine three times at 2 week intervals through intramuscular (i.m.) injection followed by electroporation at the site of delivery (Figure 2a). One week after the third injection bulk splenocytes harvested from each animal and evaluated in ELISpot assays for their ability to secrete interferon-γ (IFN-γ) after *ex vivo* exposure to peptide pools encompassing ZIKV-prME. The assay results show that splenocytes from ZIKV-prME immunised mice produced a cellular immune response after stimulation with multiple ZIKV-E peptide pools (Figure 2b). The region(s) of ZIKV-Env, which elicited the strongest cellular response(s) were evaluated by ELISpot assay in a matrix format using 22 peptide pools consisting of 15-mers (overlapping by 9 amino acids) spanning the entire ZIKV-prME protein. Several pools demonstrated elevated T cell responses, with peptide pool 15 exhibiting the highest number of spot-forming units (SFU) (Figure 2c). This matrix mapping analysis revealed a dominant prME epitope, ‘IRCIGVSNR DFVEGM’ (aa167-181). This peptide was confirmed to contain a H2-Db restricted epitope through analysis utilising the Immune Epitope Database Analysis Resource tool (http://tools.iedb.org), which˙ supports that in this haplotype the antigen is effectively processed.

Further evaluation of the cellular immunogenicity of the ZIKV-prME vaccine entailed the determination of the polyfunctional properties of CD8^+^ T cells collected 1 week after the final immunisation. The results show that the ZIKV-prME vaccination increased the proportion of bifunctional vaccine-specific T cells expressing TNF-α (tumour necrosis factor-α) and IFN-γ. Importantly, ZIKV-prME vaccination exhibited a strong ability to expand T cell functionality (Figure 2d).

In addition, comparative immune studies were performed with optimised plasmids encoding the prME sequence of either a recently identified Brazilian ZIKV strain or of the original MR766 ZIKV strain. Induction of cellular immune responses in mice immunised with either plasmid was measured 1 week after the third vaccination through IFN-γ ELISpot analysis after stimulating splenocytes with the ZIKV-prME peptide pools. The results illustrate that the T-cell responses induced by the consensus ZIKV-prME DNA vaccine construct were consistently higher than those generated by either of these two non-consensus plasmid vaccines (Supplementary Figure S1A,B). Detailed mapping analysis of the cellular responses induced by either the Brazilian or MR766 prME vaccines revealed that both vaccines induced a significant cellular response against the dominant Env-specific CTL epitope as identified in Figure 2b and c for the consensus ZIKV-prME plasmid (data not shown). The consensus immunogen consistently induced more robust responses in these T-cell assays at the same dose and was evaluated further in additional assays.

### Generation of a ZIKV recombinant envelope protein

At the onset of these studies, there were no available commercial reagents to evaluate specific anti-ZIKV immune responses. Therefore, by necessity, recombinant ZIKV-Env protein (rZIKV-E) was generated to support the assays performed in this study. To generate this reagent, a consensus ZIKV-Env sequence based on the ZIKV-prME vaccine consensus antigen was cloned into a pET30a Escherichia *coli* expression vector (Supplementary Figure S2A). The rZIKV-E antigen was produced in *E. coli* cultures, purified using nickel column chromatography and analysed using SDS-PAGE, which showed overexpressed proteins of the predicted size in lysate from rZIKV-E transfected bacteria that could be detected by western analysis using an anti-His tag antibody (Supplementary Figure S2B). The sera from mice immunised with the ZIKV-prME vaccine bound to rZIKV-Env that was used as a capture antigen in an ELISA (enzyme-linked immunosorbent assay; Supplementary Figure S2C). A commercial antibody (designated panflavivirus) that reacts to the Env protein of multiple flaviviruses, also bound to rZIKV-E. Western analysis demonstrated that immune sera from ZIKV-prME immunised mice specifically recognised rZIKV-E (Supplementary Figure S2D). These data indicate that the generated rZIKV-E reacted specifically with immune sera from ZIKV-prME vaccinated mice, thus this recombinant protein was used for further immunogenicity studies.

### Induction of functional humoral responses in C57BL/6 mice by the ZIKV-prME DNA vaccine

**Figure 3:**
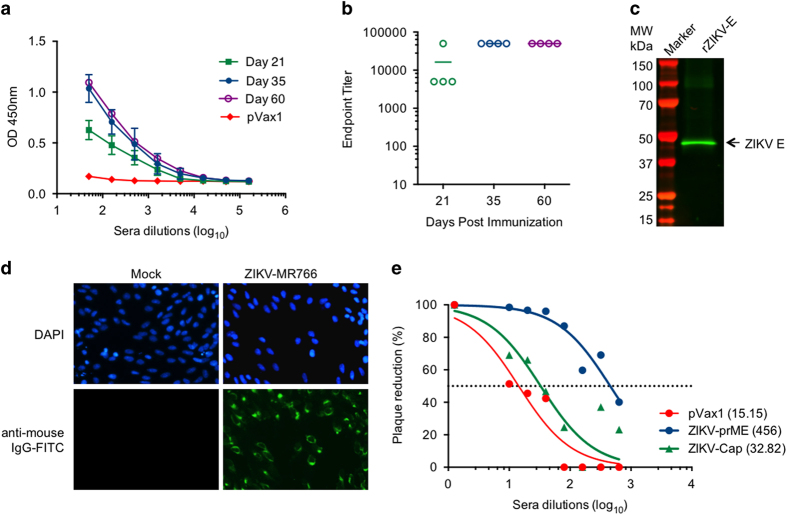
Anti-ZIKV antibody responses are induced by ZIKV-prME vaccination. (**a**) ELISA analysis measuring binding antibody production (measured by OD450 values) in immunised mice. The C57BL/6 mice (*n*=4) were immunised i.m. three times with 25 μg of ZIKV-prME plasmid or pVax1 at 2-week intervals. Binding to rZIKV-E was analysed with sera from animals at different time points (days 21, 35 and 60) post immunisation at various dilutions. The data shown are representative of at least three separate experiments. (**b**) Endpoint binding titre analysis. Differences in the anti-ZIKV endpoint titres produced in response to the ZIKV-prME immunogen were analysed in sera from immunised animals after each boost. (**c**) Western blot analysis of rZIKV-E specific antibodies induced by ZIKV-prME immunisation. The rZIKV-E protein was electrophoresed on a 12.5% SDS polyacrylamide gel and analysed by western blot analysis with pooled sera from ZIKV-prME immunised mice (day 35). Binding to rZIKV-E is indicated by the arrowhead. (**d**) Immunofluorescence analysis of ZIKV specific antibodies induced by ZIKV-prME immunisation. The Vero cells infected with either ZIKV-MR766 or mock infected were stained with pooled sera from ZIKV-prME immunised mice (day 35) followed by an anti-mouse-AF488 secondary antibody for detection. (**e**) Plaque-reduction neutralisation (PRNT) assay analysis of neutralising antibodies induced by ZIKV-prME immunisation. The serum samples from the ZIKV-prME immunised mice were tested for their ability to neutralise ZIKV infectivity *in vitro.* PRNT_50_ was defined as the serum dilution factor that could inhibit 50% of the input virus. The values in parentheses indicate the PRNT_50_. Control ZIKV-Cap (DNA vaccine expressing the ZIKV capsid protein) and pVax1 sera were used as negative controls. ZIKV-prME, precursor membrane and Env of Zika virus.

The ability of the consensus ZIKV-prME vaccine to induce humoral immune responses in mice was evaluated. Groups of four C57BL/6 mice were immunised intramuscularly (i.m.) through electroporation-mediated delivery three times at 2-week intervals with 25 μg of either the empty control pVax1 or the consensus ZIKV-prME vaccine plasmids. Sera was obtained from each immunized mouse and tested by ELISA for ZIKV-specific IgG responses using immobilised rZIKV-E as the capture antigen. A significant increase in anti-ZIKV-specific IgG was observed on day 21 with a further boost in the sera IgG levels noted on day 35 (Figure 3a). Day 60 sera from vaccinated animals show that elevated ZIKV-specific antibody responses were maintained long term following the final boost. Most importantly, sera from vaccinated mice contained very high levels of rZIKV-E-specific antibodies as indicated by the endpoint titres (Figure 3b). Additional assessment of the specificity of the vaccine-induced antibodies was performed by screening pooled sera from ZIKV-prME plasmid inoculated mice for its ability to detect rZIKV-E by western analysis (Figure 3c) and to stain ZIKV (MR766 strain)-infected cells in an immunofluorescence assay (Figure 3d). The results from both these analyses confirmed specificity of the vaccine-induced humoral responses.

Furthermore, ZIKV-specific binding antibody responses were also assessed in mice immunised with plasmids encoding the prME sequences from a Brazilian strain and the MR766 strain described above. Day 35 (1 week after third immunisation) sera from pVax1- and both non-consensus vaccine-immunised mice were analysed by ELISA for binding to rZIKV-E. This analysis indicates that both MR766 and Brazil vaccine plasmids induced significant antibody binding, and that immunisation with the consensus ZIKV-prME DNA vaccine generates an effective humoral response against rZIKV-E (Supplementary Figure S1C, D).

A plaque reduction neutraliszation test (PRNT) assay was performed on pooled day 35 sera from mice immunised with either the control pVax1 plasmid, the consensus ZIKV-prME plasmid vaccine or a consensus ZIKV-C (capsid) plasmid vaccine. The PRNT assay used was a method adapted from a previously described technique for analysing dengue virus, West Nile virus and other flaviviruses.^16^ As shown in Figure 3e, ZIKV-prME vaccination yielded significant neutralisation response with anti-ZIKV reciprocal PRNT_50_ dilution titres (inverse of the serum dilution at which 50% of the control ZIKV infection was inhibited) of 456±5, whereas the PRNT50 of sera from ZIKV-Cap DNA vaccine immunized mice was 33±6 which was minimally higher than that of sera from pVax1 control plasmid vaccinated animals (titre=15±2).

### Immune responses and protection against ZIKV in mice lacking the type I interferon receptor (IFNAR^−^/^−^) following immunisation with the ZIKV-prME DNA vaccine

Mechanisms of ZIKV-induced disease and immunity are poorly defined, and the protective versus the hypothetical pathogenic nature of the immune response to ZIKV infection is as yet unclear.^17^ Most strains of mice are resistant to ZIKV infection, however, mice lacking IFN-α/β receptor (IFNAR^−^/^−^) were found to be susceptible to infection and disease with most succumbing within 6–7 days post challenge.^18^ The ability of the consensus ZIKV-prME plasmid vaccine to induce cellular and humoral immune responses in this mouse strain was investigated. Five to six week old female IFNAR^−^/^−^ mice (*n*=4) were immunised i.m., with electroporation-mediated delivery, three times at 2-week intervals with either the control pVax1 plasmid or ZIKV-prME vaccine plasmid vaccine. The serum was collected from immunised mice at days 0, 14, 21, and 35, and splenocytes were harvested from mice 1 week following the final immunisation (day 35). The splenocytes from vaccine-immunised mice produced a clear cellular immune response as indicated by levels of SFU per 10^6^ cells in an ELISpot assay (Supplementary Figure S3A). The results from ELISA analysis, using rZIKV-E as a capture antigen, show detectable anti-ZIKV serum IgG by day 14 (titres of ~1:1,000) and these levels were boosted with subsequent vaccinations with binding antibody titres reaching at least 1:100,000 (Supplementary Figure S3B,C). By comparison, the PRNT_50_ titre for the day 35 postimmunisation samples was 1:60 (data not shown). The results indicate that IFNAR^−^/^−^ mice immunised with the consensus ZIKV-prME vaccine are capable of generating anti-ZIKV cellular and humoral immune responses supporting further study in this model of putative vaccine effects in a pathogenic challenge.

### ZIKV-specific functional cellular and humoral responses elicited by the ZIKV-prME DNA vaccine in non-human primates

**Figure 4:**
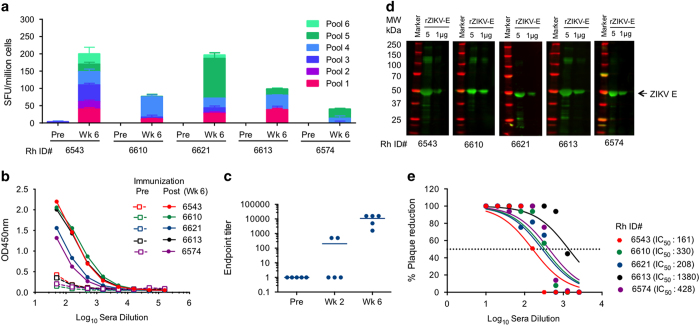
Induction of ZIKV specific cellular immune responses following ZIKV-prME vaccination of non-human primates (NHPs). (**a**) ELISpot analysis measuring IFN-γ secretion in peripheral blood mononuclear cells (PBMCs) in response to ZIKV-prME immunisation. Rhesus macaques were immunised intradermally with 2 mg of ZIKV-prME plasmid at weeks 0 and 4 administered as 1 mg at each of two sites, with immunisation immediately followed by intradermal electroporation. PBMCs were isolated pre-immunisation and at week 6 and were used for the ELISPOT assay to detect IFN-γ-secreting cells in response to stimulation with ZIKV-prME peptides as described in the ‘Materials and Methods’ section. The number of IFN-γ producing cells obtained per million PBMCs against six peptide pools encompassing the entire prME protein is shown. The values represent mean responses in each group (*n*=5)±s.e.m. (**b**) Detection of ZIKV-prME-specific antibody responses following DNA vaccination. Anti-ZIKV IgG antibodies were measured pre-immunisation and at week 6 by ELISA. (**c**) Endpoint ELISA titres for anti ZIKV-Env antibodies are shown following the first and second immunisations. (**d**) Western blot analysis using week 6 RM immune sera demonstrated binding to recombinant Env protein. (**e**) PRNT activity of serum from RM immunised with ZIKV-prME. Pre-immunisation and week 6 immune sera from individual monkeys were tested by plaque-reduction neutralisation (PRNT) assay for their ability to neutralise ZIKV infectivity *in vitro*. PRNT_50_ was defined as the serum dilution factor that could inhibit 50% of the input virus. Calculated (PRNT_50_) values are listed for each monkey. IFN, interferon; ZIKV-prME, precursor membrane and Env of ZIKV.

NHPs were immunised by intradermal immunisation using intradermal electroporation, based on recent studies showing potent immune responses in a intradermal format.^19,20^ Rhesus macaques (RM; *n*=5/group) were administered 2.0 mg of vaccine plasmid intradermally with electroporation, with each animal vaccinated twice 4 weeks apart. The sera and peripheral blood mononuclear cells (PBMCs) were collected at day 0 (pre-immunisation) and week 6 (2 weeks post second immunisation). ELISpot analysis of pre-immunisation and week 6 PBMCs *ex vivo* stimulated with the ZIKV-prME peptide pools showed that ZIKV-prME immunisation induced robust anti-ZIKV T cell responses in RM (Figure 4a).

Specific anti-ZIKV antibody responses in sera from vaccinated RM were assessed by ELISA. At week 6, rZIKV-E-specific binding antibodies were detectable in animals vaccinated with ZIKV-prME (Figure 4b). Endpoint titres were determined for each animal at week 2 (after 1 immunisation) and week 6 (after 2 immunisations; Figure 4c). The ELISA results were confirmed by western blot analysis using RM sera from the individual vaccinated animals (Figure 4d). The neutralisation activity of the antibodies generated in RM at week 6 was evaluated by a PRNT_50_ assay. All the vaccinated monkeys had significant neutralisation activity with anti-ZIKV reciprocal PRNT_50_ dilution titres ranging from 161 to 1380 (average 501±224 standard error of the mean; Figure 4e). PRNT titres did not directly correlate with ELISA titre (data not shown).

The ability of the immune sera from vaccinated RM to block ZIKV infection of Vero cells, neuroblastoma (SK-N-SH) or neural progenitor (U-87MG) cells *in vitro* was examined by IFA. ZIKV strains (MR766 or PR209) were pre-incubated in sera or diluted RM-immune sera and added to monolayers of each cell type. Four days post infection, ZIKV-positive cells were identified by IFA using pan flavivirus antibody (Supplementary Figure S4A–C) and ZIKV-positive cells were quantified (Supplementary Figure S4B–D). The sera from ZIKA-prME vaccinated RM inhibited the ZIKV infection in each cell type tested.

### Protection against ZIKV infection and disease in IFNAR^−^/^−^ mice following ZIKV-prME immunisation

In exploratory studies, 5–6-week-old IFNAR^(−^/^−)^ mice (*n*=10) were challenged with 1×10^6^ plaque-forming units (PFU) of the ZIKV-PR209 isolate, administered by either subcutaneous (s.c.); intraperitoneal (i.p.); intracranial (i.c); or intravenous (i.v.) routes. After the challenge, all the animals were monitored for clinical signs of infection, which included routine measurement of body weight as well as inspection for other signs of a moribund condition such as hind limb weakness and paralysis. No change in the general appearance of the mice was observed during the first 4 days after inoculation. However, after the fourth day, the mice in each of the groups demonstrated reduced overall activity, decreased mobility and a hunched posture often accompanied by hind-limb weakness, decreased water intake and obvious weight loss. The animals succumbed to the infection between day 6 and day 8 regardless of the route of viral challenge (Supplementary Figure S5A–E). On the basis of these data, the subsequent studies to evaluate ZIKV-prME-mediated protection in this model used the s.c. route for challenge.

**Figure 5:**
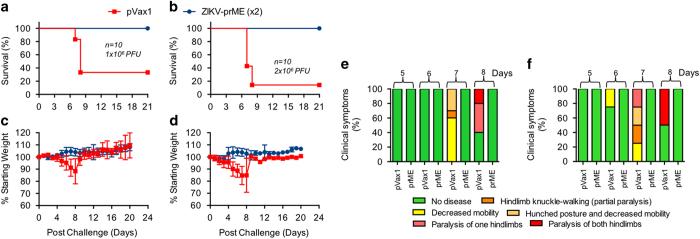
Survival data for immunised mice lacking the type I interferon α, β receptor following ZIKV infection. Mice were immunised twice with 25 μg of the ZIKV-prME DNA vaccine at 2-week intervals and challenged with ZIKV-PR209 virus 1 week after the second immunisation with 1×10^6^ plaque-forming units (PFU; **a**) or 2×10^6^ PFU (**b**) viral dose. Weight change (**c**,**d**) and clinical scores (**e**,**f**) for animals in (**a** and **b**), respectively are indicated. The designation for the clinical scores is as follows: 1: no disease, 2: decreased mobility; 3: hunched posture and decreased mobility; 4: hind limb knuckle walking (partial paralysis); 5: paralysis of one hind limb; and 6: paralysis of both hind limbs. The data reflect the results from two independent experiments with 10 mice per group per experiment. ZIKV-prME, precursor membrane and Env of ZIKV.

The protective efficacy of the ZIKV-prME vaccine was next evaluated in this IFNAR^−/−^ mouse model. Two groups of mice (*n*=10) were immunised (25 μg of vaccine) by the i.m. route, through electroporation-mediated delivery with the ZIKV-prME vaccine. Also, two groups of 10 mice were immunised by the i.m. route through electroporation-mediated delivery with the control pVax1 vector. The immunisations were performed two times, two weeks apart, and all the animals were challenged on day 21 (1 week post second immunisation). One set of control and vaccinated mice received 1×10^6^ PFU of ZIKV-PR209 by the s.c. route and the other set of each group were challenged with a total of 2×10^6^ PFU ZIKV-PR209 by the s.c. route. At 3 weeks post challenge, 100% of all ZIKV-prME vaccinated animals survived, whereas only 30% of the single- or 10% of double-dose challenged controls survived (Figures 5a,b). In all the challenges, the vaccinated animals were without signs of disease including no evidence of weight loss (Figures 5c,d). The infection of control mice with ZIKV-PR209 virus produced a marked decrease in body weight along with decreased mobility, hunched posture, hind-limb knuckle walking and/or paralysis of one or both hind limbs (Figures 5e,f).

**Figure 6:**
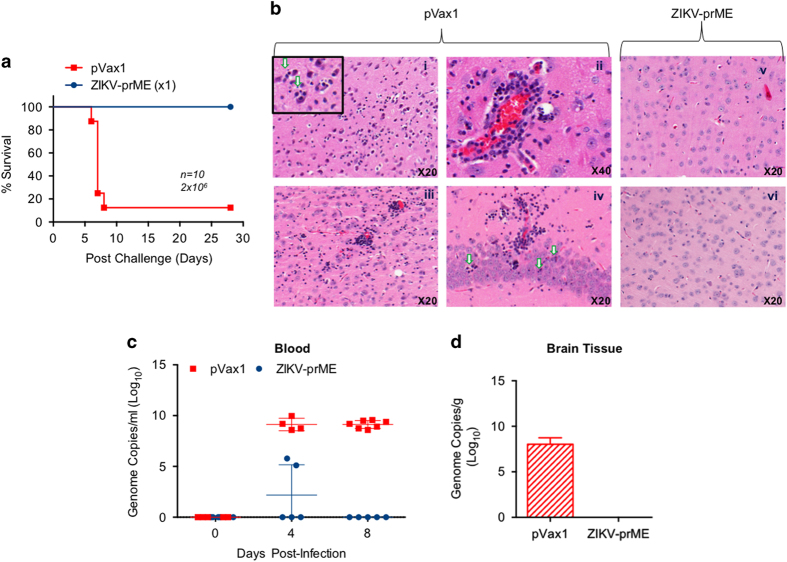
Single immunisation with the ZIKV-prME vaccine provides protection against ZIKV challenge in mice lacking the type I interferon α, β receptor. Mice were immunised once and challenged with 2×10^6^ plaque-forming units of ZIKV-PR209, two weeks post immunisation. The survival curves depict 10 mice per group per experiment (**a**). The ZIKV-prME vaccine prevented ZIKV-induced neurological abnormalities in the mouse brain (**b**). Brain sections from pVax1 and ZIKV-prME vaccinated groups were collected 7–8 days after challenge and stained with H&E (haematoxylin and eosin) for histology. The sections taken from representative, unprotected pVax1 control animals shows pathology. (i): nuclear fragments within neuropils of the cerebral cortex (inset shows higher magnification and arrows to highlight nuclear fragments); (ii): perivascular cuffing of vessels within the cortex, lymphocyte infiltration and degenerating cells; (iii): perivascular cuffing, cellular degeneration and nuclear fragments within the cerebral cortex; and (iv): degenerating neurons within the hippocampus (arrows). An example of normal tissue from ZIKV-prME vaccinated mice appeared to be within normal limits (v and vi). (**c**) Levels of ZIKV RNA in the plasma samples from mice following vaccination and viral challenge at the indicated day post infection. The results are indicated as the genome equivalents per millilitre of plasma. (**d**) Levels of ZIKV-RNA in the brain tissues were analysed at day 28 post infection. The results are indicated as the genome equivalent per gram of tissue. ZIKV-prME, precursor membrane and Env of Zika virus.

The potential ability of a single immunisation with the ZIKV-prME DNA vaccine to protect IFNAR^−^/^−^ mice from ZIKV challenge was evaluated. Groups of 10 mice were immunised i.m. with electroporation once with either control plasmid or ZIKV-prME vaccine and challenged 2 weeks later with a total dose of 2×10^6^ PFU ZIKV-PR209. Three weeks post challenge, 100% of the ZIKV-prME vaccinated animals survived, whereas only 10% of the control animals survived (Figure 6a). To determine gross histopathological changes, brain tissue was sectioned into 5 μm-thick sagittal sections, stained for nuclear structures and counterstained for cytoplasmic structures using eosin (Figure 6b). The mice were killed at day 7 or 8 post challenge for the analysis of histology and viral load. The ZIKV infection caused severe brain pathology in the mice. The unvaccinated control (pVax1) mice brain sections showed nuclear fragments within neutrophils (Figures 6b–i); perivascular cuffing of vessels within the cortex, lymphocyte infiltration and degenerating cells of the cerebral cortex (Figure 6b-ii and iii) and degenerating neurons within the hippocampus (Figure 6b-iv). In contrast, the ZIKV-prME vaccinated animals presented with normal histopathology in brain tissues (Figures 6b-v and vi) supporting that protective responses induced by immunisation with the synthetic ZIKA-prME vaccine could limit viral-induced disease in the brain. This observation demonstrates the potential for vaccination to protect the brain in this model. Consistent with the amelioration of body weight loss and mobility impairment in vaccinated mice following ZIKV challenge, a significantly lower viral load was noted in the blood (Figure 6c) and brain (Figure 6d) of the ZIKV-prME vaccinated animals compared with viral challenged pVax1 vaccinated animals in the high (2×10^6^ PFU) dose challenge groups. Taken together, these data illustrate that ZIKV-prME DNA vaccine-mediated immune responses can protect mice against ZIKV challenge.

### Passive transfer of anti-ZIKV immune sera protects mice against ZIKV infection

**Figure 7:**
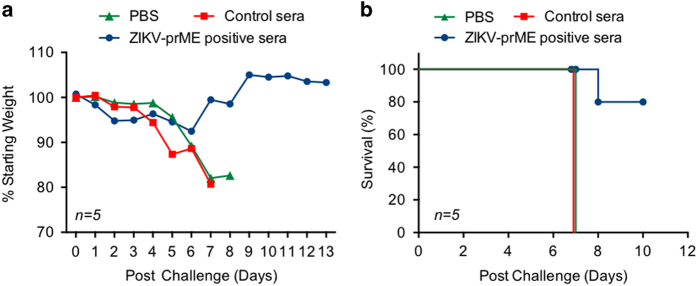
Protection of mice lacking the type I interferon α, β receptor following passive transfer of anti-ZIKV immune sera following ZIKV challenge. Pooled NHP anti-ZIKV immune sera, titred for anti-ZIKA virus IgG, was administered i.p. (150 μl/mouse) to mice 1 day after s.c. challenge with a ZIKV (10^6^ plaque-forming units per mouse). As a control, normal monkey sera and phosphate-buffered saline (PBS) were administered (150 μl/mouse) to age-matched mice as controls. (**a**) Mouse weight change during the course of infection and treatment. Each point represents the mean and standard error of the calculated percent pre-challenge (day 0) weight for each mouse. (**b**) Survival of mice following administration of the NHP immune sera. ZIKV-prME, precursor membrane and Env of ZIKV.

Next, we tested whether transfer of immune sera from ZIKV-prME vaccinated RM could prevent ZIKV-mediated pathogenesis in IFNAR^−^/^−^ mice. To this end, 150 μg equivalent IgG (PRNT_50_≈1/160) from week 6 RM were adoptively transferred into IFNAR^−^/^−^ mice 1 day after the ZIKV viral challenge. Two groups of control mice were included, one group receiving pre-immune sera from RM and the other group receiving phosphate-buffered saline (PBS). The mice that received PBS or control sera lost 15 to 25% of their original body weight during the course of infection, and all died 6–8 days post infection. When vaccine immune sera from RMs were transferred to infection-susceptible mice, the animals lost weight on day 3 and 4, but subsequently regained it beginning on day 5 and 80% ultimately survived infectious challenge (Figure 7a) demonstrating the ability of the NHP sera transfer to confer protection against clinical manifestations of ZIKV infection following viral challenge (Figure 7b). In repeated experiments performed to evaluate the efficacy of immune serum transfer in protection against challenge with ZIKV, the survival among ZIKV-prME immune sera recipients ranged from 80 to 100%. These studies show that anti-ZIKV vaccine immune sera had the ability to confer significant protection against ZIKV infection in the absence of an acquired adaptive anti-ZIKV immune response.

## Discussion

Serious concerns have been raised by the recent spread of ZIKV and its associated pathogenesis in humans. Currently, there are no licensed vaccines or therapeutics for this emerging infectious agent. Very recently, a collection of experimental ZIKV vaccines have been shown to lower viral load post challenge in non-pathogenic animal infection models.^21,22^ These data are encouraging. In this regard, it is important to examine additional novel vaccine approaches targeting ZIKA in additional models that similar to susceptible humans, might show disease. Here we evaluated a synthetic DNA vaccine, designed to express a novel consensus ZIKV-prM and E antigen, for immunogenicity following electroporation-enhanced immunisation in mice and non-human primates. We observed that ZIKV-prME DNA vaccination was immunogenic and generated antigen-specific T cells and binding and neutralising antibodies in both mice and NHPs. Uniquely, the NHPs were immunised with ZIKV-prME through electroporation by the intradermal route, which uses lower voltage and a smaller transfection area than i.m. electroporation, as we have recently described.^23^ Further study of such approaches may provide advantages in clinical settings.

The ZIKV-prME consensus construct includes a designed change of the potential NXS/T motif, which removes a putative glycosylation site. Deletion of glycosylation at this site has been correlated with improved binding of EDE1 type bnAbs (broadly neutralising antibodies) against ZIKV-E protein.^24^ The antibody responses induced by the consensus ZIKV-prME appear as robust or in some cases superior in magnitude to those elicited by similarly developed MR766 ZIKV-prME and Brazil ZIKV-prME vaccines. These constructs were sequence matched with the original ZIKV-MR766 isolate or a recently circulating ZIKV strain from Brazil, respectively. While supportive, further study will provide more insight into the effects of such incorporated designed changes on induced immune responses.

As there are few pathogenic challenge models for ZIKV, we compared the putative protective nature of the immune responses of the ZIKV-prME vaccine in C57BL/6 and IFNAR^−/−^ mice. Both strains of mice responded with a robust humoral immune response when immunised with ZIKV-prME. T-cell responses were also induced, but appear to be more robust in wild-type C57BL/6 compared with those induced in the IFNAR^−^/^−^ animals, supporting a partial defect in innate to adaptive immunity transition as expected owing to the knock-out phenotype in the mouse. However, based on the induction of antigen specific immunity, the model was useful for evaluation of the impact of the vaccine on both infection and pathogenesis. A single vaccination with ZIKV-prME in IFNAR^−^/^−^ mice was protective against disease and death in this model, including protection of neuro-pathogenesis. Flavivirus-neutralising antibodies directed against the Env antigen are thought to have a key role in protection against disease, an idea supported directly by passive antibody transfer experiments in animal models and indirectly by epidemiological data from prospective studies in geographical areas that are prone to mosquito-borne viral infections.^3,7,8^ Although immunisation of IFNAR^−^/^−^ mice with the ZIKV-prME DNA vaccine as well as serum transfer from immunised NHPs were protective in this murine model, the IFNAR^−^/^−^ vaccinated as opposed to serum-transferred mice exhibited greater control of weight loss as an indication of control of pathogenesis. Although additional studies are needed, this result potentially suggests a role for the T-cell response in this aspect of protection in this model. In addition, we observed that control IFNAR^−^/^−^ mice who recovered from challenge remain viral positive by PCR for at least several weeks, suggesting an additional benefit of vaccination in limiting potential sexual or vector borne transmission. Our study extends prior findings and supports the potential of vaccination and, in this case this synthetic DNA vaccination, to impact prevention of disease in a susceptible host.

## Materials and Methods

### Cells, virus and animals

Human embryonic kidney 293T (American Type Culture Collection (ATCC) #CRL-N268, Manassas, VA, USA) and Vero CCL-81 (ATCC #CCL-81) cells were maintained in DMEM (Dulbecco's modified Eagle's medium; Gibco-Invitrogen, Carlsbad, CA, USA) supplemented with 10% fetal bovine serum (FBS) and 1% penicillin and streptomycin and passaged upon confluence. Both ZIKV virus strains MR766 (a kind gift from Dr Susan Weiss) and PR209 (Bioqual, MD) were amplified in Vero cells and stocks were titred by standard plaque assay on Vero cells. Five- to six-week-old female C57BL/6 (The Jackson Laboratory, Bar Harbor, ME, USA) and IFNAR^−^/^−^ (MMRRC repository-The Jackson Laboratory) mice were housed and treated/vaccinated in a temperature-controlled, light-cycled facility in accordance with the National Institutes of Health, Wistar and the Public Health Agency of Canada IACUC (Institutional Animal Care and Use Committee) guidelines.

The RMs were housed and treated/vaccinated at Bioqual, MD, USA. This study was carried out in strict accordance with the recommendations described in the Guide for the Care and Use of Laboratory Animals of the NIH, the Office of Animal Welfare, and the U.S. Department of Agriculture. All animal immunisation work was approved by the Bioqual Animal Care and Use Committee (IACUC). Bioqual is accredited by the American Association for Accreditation of Laboratory Animal Care. All the procedures were carried out under ketamine anaesthesia by trained personnel under the supervision of veterinary staff, and all the efforts were made to protect the welfare of the animals and to minimise animal suffering in accordance with the ‘Weatherall report for the use of non-human primates’ recommendations. The animals were housed in adjoining individual primate cages allowing social interactions, under controlled conditions of humidity, temperature and light (12 h light/12 h dark cycles). Food and water were available *ad libitum*. The animals were monitored twice daily and fed commercial monkey chow, treats and fruits twice daily by trained personnel.

### Construction of ZIKV-prME DNA vaccine

The ZIKV-prME plasmid DNA constructs encodes full-length precursor of membrane (prM) plus Env (E) and a construct encoding a consensus Capsid proteins were synthesised. A consensus strategy was used and the consensus sequences were determined by the alignment of current ZIKV prME protein sequences. The vaccine insert was genetically optimised (i.e., codon and RNA optimisation) for enhanced expression in humans and an IgE leader sequence was added to facilitate expression. The construct was synthesised commercially (Genscript, NJ, USA), and then subcloned into a modified pVax1 expression vector under the control of the cytomegalovirus immediate-early promoter as described before.^25^ The final construct is named ZIKV-prME vaccine and the control plasmid backbone is pVax1. In addition, a number of other matched DNA constructs encoding the prM and E genes from MR766 (DQ859059.1) and a 2016 Brazilian (AMA12084.1) outbreak strain were also designed, for further evaluation. Large-scale amplifications of DNA constructs were carried out by Inovio Pharmaceuticals Inc. (Plymouth Meeting, PA, USA) and purified plasmid DNA was formulated in water for immunisations. The size of the DNA inserts was confirmed via agarose gel electrophoresis. Phylogenetic analysis was performed by multiple alignment with ClustalW using MEGA version 5 software.^25^

### DNA immunisations and electroporation-mediated delivery enhancement

Female C57BL/6 mice (6–8 weeks old) and IFNAR^−^/^−^ mice (5–6 weeks old) were immunised with 25 μg of DNA in a total volume of 20 or 30 μl of water delivered into the tibialis anterior muscle with *in vivo* electroporation delivery. *In vivo* electroporation was delivered with the CELLECTRA adaptive constant current electroporation device (Inovio Pharmaceuticals) at the same site immediately following DNA injection. A three-pronged CELLECTRA minimally invasive device was inserted ~2 mm into the muscle. Square-wave pulses were delivered through a triangular three-electrode array consisting of 26-gauge solid stainless steel electrodes and two constant current pulses of 0.1 Amps were delivered for 52 msec/pulse separated by a 1 s delay. Further protocols for the use of electroporation have been previously described in detail.^26^ The mice were immunised three times at 2-week intervals and killed 1 week after the final immunisation. Blood and splenocytes were collected after each immunisation for the analysis of cellular and humoral immune responses.^25^ Rhesus macaque immunogenicity studies: five rhesus macaques were immunised intradermally at two sites two times at 4-week intervals with 2 mg ZIKV-prME vaccine. Electroporation was delivered at 0.2 Amps immediately using the same device described for mouse immunisations.

### Western blot analysis

For *in vitro* expression studies, transfections were performed using the GeneJammer reagent, following the manufacturer’s protocols (Agilent, Santa Clara, CA, USA). Briefly, the cells were grown to 50% confluence in a 35 mm dish and transfected with 1 μg of ZIKV-prME vaccine. The cells were collected 2 days after transfection, washed twice with PBS and lysed with cell lysis buffer (Cell Signaling Technology, Danvers, MA, USA). Western Blot was used to verify the expression of the ZIKV-prME protein from the harvested cell lysate and the immune specificity of the mouse and RM serum through the use of either pan-flavivirus antibody or immune sera from the ZIKV-prME vaccinated mice, as described previously.^25^ In brief, 3–12% Bis-Tris NuPAGE gels (Life Technologies, Carlsbad, CA, USA) were loaded with 5 μg or 1 μg of ZIKV Env recombinant protein (rZIKV-E); transfected cell lysates or supernatant and the Odyssey protein Molecular Weight Marker (Product # 928-40000). The gels were run at 200 V for 50 min in MOPS buffer. The proteins were transferred onto nitrocellulose membranes using the iBlot 2 Gel Transfer Device (Life Technologies). The membranes were blocked in PBS Odyssey blocking buffer (LI-COR Biosciences, Lincoln, NE, USA) for 1 h at room temperature. To detect vaccine expression, the anti-Flavivirus group antigen (MAB10216-Clone D1-4G2-4-15) antibody was diluted 1:500 and the immune serum from mice and RM was diluted 1:50 in Odyssey blocking buffer with 0.2% Tween 20 (Bio-Rad, Hercules, CA, USA) and incubated with the membranes overnight at 4 °C. The membranes were washed with PBST and then incubated with the appropriate secondary antibody (goat anti-mouse IRDye680CW; LI-COR Biosciences) for mouse serum and flavivirus antibody; and goat anti-human IRDye800CW (LI-COR Biosciences) for RM sera at 1:15,000 dilution for mouse sera for 1 h at room temperature. After washing, the membranes were imaged on the Odyssey infrared imager (LI-COR Biosciences).

### Immunofluorescence assays

For the immunofluorescence assay, the cells were grown on coverslips and transfected with 5 μg of ZIKV-prME vaccine. Two days after transfection, the cells were fixed with 4% paraformaldehyde for 15 min. Nonspecific binding was then blocked with normal goat serum diluted in PBS at room temperature for 1 h. The slides were then washed in PBS for 5 min and subsequently incubated with sera from immunised mice or RM at a 1:100 dilutions overnight at 4 °C. The slides were washed as described above and incubated with appropriate secondary antibody (goat anti-mouse IgG-AF488; for mouse serum and goat anti-human IgG-AF488 for RM serum; Sigma, St Louis, MO, USA) at 1:200 dilutions at room temperature for 1 h. After washing, Flouroshield mounting media with DAPI (Abcam, Cambridge, MA, USA) was added to stain the nuclei of all cells. After which, coverslips were mounted and the slides were observed under a microscope (EVOS Cell Imaging Systems; Life Technologies).^25^. In addition, Vero, SK-N-SH or U87-MB cells were grown on four-chamber tissue culture treated glass slides and infected at MOI of 0.01 with ZIKV-MR766 or PR209 that were preincubated with/without RM immune sera (1:200), and stained at 4 days post ZIKV infection using pan-flavivirus antibody as described.^17^

### Histopathology analysis

For histopathology, formalin-fixed, paraffin-embedded brain tissue was sectioned into 5 μm thick sagittal sections, placed on Superfrost microscope slides (Fisher Scientific, Hampton, NH, USA) and backed at 37 °C overnight. The sections were deparaffinised using two changes of xylene and rehydrated by immersing in 100%, 90% and then 70% ethanol. The sections were stained for nuclear structures using Harris haematoxylin (Surgipath, Buffalo Grove, IL, USA) for 2 min followed by differentiation in 1% acid alcohol (Surgipath) and treatment with Scott’s tap water for 2 min. Subsequently, the sections were counterstained for cytoplasmic structures using eosin (Surgipath) for 2 min. The slides were dehydrated with 70%, 90% and 100% ethanol, cleared in xylene and mounted using Permount (Fisher Scientific).

### Splenocyte and PBMC isolation

Single-cell suspensions of splenocytes were prepared from all the mice. Briefly, the spleens from mice were collected individually in 5 ml of RPMI 1640 supplemented with 10% FBS (R10), then processed with a Stomacher 80 paddle blender (A.J. Seward and Co. Ltd.) for 30 s on high speed. The processed spleen samples were filtered through 45 mm nylon filters and then centrifuged at 1,500*g* for 10 min at 4 °C. The cell pellets were resuspended in 5 ml of ACK (ammonium–chloride–potassium) lysis buffer (Life Technologies) for 5 min at room temperature, and PBS was then added to stop the reaction. The samples were again centrifuged at 1,500*g* for 10 min at 4 °C. The cell pellets were resuspended in R10 and then passed through a 45 mm nylon filter before use in ELISpot assay and flow cytometric analysis.^25^ For RM, blood (20 ml at each time point) was collected in EDTA tubes and the PBMCs were isolated using a standard Ficoll-hypaque procedure with Accuspin tubes (Sigma-Aldrich, St. Louis, MO, USA). Five millilitres of blood was also collected into sera tubes at each time point for sera isolation.

### Flow cytometry and intracellular cytokine staining assay

The splenocytes were added to a 96-well plate (2×10^6^/well) and were stimulated with ZIKV-prME pooled peptides for 5 h at 37 °C/5% CO_2_ in the presence of Protein Transport Inhibitor Cocktail (brefeldin A and monensin; eBioscience, San Diego, CA, USA). The cell stimulation cocktail (plus protein transport inhibitors; PMA (phorbol 12-myristate 13-acetate), ionomycin, brefeldin A and monensin; eBioscience) was used as a positive control and R10 media as the negative control. All the cells were then stained for surface and intracellular proteins as described by the manufacturer’s instructions (BD Biosciences, San Diego, CA, USA). Briefly, the cells were washed in FACS buffer (PBS containing 0.1% sodium azide and 1% FBS) before surface staining with flourochrome-conjugated antibodies. The cells were washed with FACS buffer, fixed and permeabilised using the BD Cytofix/Ctyoperm TM (BD Biosciences) according to the manufacturer’s protocol followed by intracellular staining. The following antibodies were used for surface staining: LIVE/DEAD Fixable Violet Dead Cell stain kit (Invitrogen), CD19 (V450; clone 1D3; BD Biosciences) CD4 (FITC; clone RM4-5; eBioscience), CD8 (APC-Cy7; clone 53-6.7; BD Biosciences); CD44 (BV711; clone IM7; BioLegend, San Diego, CA, USA). For intracellular staining, the following antibodies were used: IFN-γ (APC; clone XMG1.2; BioLegend), TNF-α (PE; clone MP6-XT22; eBioscience, San Diego, CA, USA), CD3 (PerCP/Cy5.5; clone 145-2C11; BioLegend); IL-2 (PeCy7; clone JES6-SH4; eBioscience). All the data were collected using a LSRII flow cytometer (BD Biosciences) and analysed using FlowJo software (Tree Star, Ashland, OR, USA).

### ELISpot assay

Briefly, 96-well ELISpot plates (Millipore, Billerica, MA, USA) were coated with anti-mouse IFN-γ capture Ab (R&D Systems, Minneapolis, MN, USA) and incubated overnight at 4 °C. The following day, the plates were washed with PBS and blocked for 2 h with PBST+1% BSA. Two hundred thousand splenocytes from immunised mice were added to each well and incubated overnight at 37 °C in 5% CO_2_ in the presence of media alone (negative control), media with PMA/ionomycin (positive control) or media with peptide pools (1 μg/ml) consisting of 15-mers overlapping by nine amino acids and spanning the length of the ZIKV prME protein (Genscript, Piscataway, NJ, USA). After 24 h, the cells were washed and then incubated overnight at 4 °C with biotinylated anti-mouse IFN-γ Ab (R&D Systems, Minneapolis, MN, USA). Streptavidin–alkaline phosphatase (R&D Systems) was added to each well after washing and then incubated for 2 h at room temperature. The plate was washed, and then 5-bromo-4-chloro-3′-indolylphosphate *p*-toluidine salt and nitro blue tetrazolium chloride (chromogen colour reagent; R&D Systems) was added. Last, the plates were rinsed with distilled water, dried at room temperature and SFU were quantified by an automated ELISpot reader (CTL Limited, Shaker Heights, OH, USA), and the raw values were normalised to SFU per million splenocytes. For RM samples, the ELISPOT^PRO^ for monkey IFN-γ kit (MABTECH, Cincinnati, OH, USA) was used as described by the manufacturer; two hundred thousand PBMCs were stimulated with peptide pools; and the plates were washed and spots were developed and counted as described before.^25^

### Humoral immune response: antibody-binding ELISA

An ELISA was used to determine the titres of mouse and RM sera as previously described.^25^ Briefly, 1 μg of purified rZIKV-E protein was used to coat 96-well microtiter plates (Nalgene Nunc International, Naperville, IL, USA) at 4 °C overnight. After blocking with 10% FBS in PBS for at least an hour, the plates were washed four times with 0.05% PBST (Tween20 in PBS). Serum samples from immunised mice and RMs were serially diluted in 1% FBS, added to the plates, then incubated for 1 h at room temperature. The plates were again washed four times in 0.05% PBST, then incubated with HRP-conjugated anti-mouse IgG (Sigma) at a 1:35,000 dilution for mouse sera for 1 h at room temperature. For RM sera, anti-monkey IgG HRP (Southern Biotech, Birmingham, AL, USA) was used at a 1:5,000 dilutions for 1 h at room temperature. The bound enzyme was detected by adding SIGMAFAST OPD (*o*-phenylenediamine dihydrochloride) substrate solution according to the manufacturer’s instructions (Sigma-Aldrich, St. Louis, MO, USA). The reaction was stopped after 15 min with the addition of 1 N H_2_SO_4_. The optical density at 450 nm was read on a Synergy plate reader. All the mouse and RM serum samples were assayed in duplicate. Endpoint titres were determined using the method described by Frey *et al.*^27^

### Neutralisation (PRNT_50_) assay

The PRNT involving MR766 and Vero cells was described previously.^28^ Briefly, heat-inactivated mouse or RM sera were serially diluted in serum-free DMEM (1:10 to 1: 1280) and incubated with an equal volume of ZIKV MR766 (100 PFU) at 37 °C for 2 h. The mixtures were added to the confluent layers of Vero cells and left at 37 °C for adsorption for 2 h. A 2× DMEM media:soft-agar (1:1) overlay was added over cells and the plate was incubated for 5 days at 37 °C. The agar overlay was removed and the cells were fixed with 4% paraformaldehyde, washed with 1× PBS, stained with crystal violet solution, washed with 1× PBS and the plates were left to dry. The plaques in assays done in 24-well plates were scanned with an automated Immunospot reader (CTL Limited), and the plaques in sample wells and in negative control (DMEM only) and positive control (100 PFU MR766 ZIKV virus only) wells were counted using the automated software provided with the ELISpot reader. The percentage plaque reduction was calculated as follows: % reduction=100×{1−(average number of plaques for each dilution/average number of plaques in positive control wells)}. GraphPad Prism software was used to perform nonlinear regression analysis of % plaque reduction versus a log transformation of each individual serum dilution to facilitate linear interpolation of actual 50% PRNT titres at peak post vaccination response. The medians and interquartile ranges at 50% neutralisation were calculated for each neutralisation target overall and by vaccine treatment group; the geometric mean titres were also calculated. The titres represent the reciprocal of the highest dilution resulting in a 50% reduction in the number of plaques.

### ZIKV challenge studies in IFNAR^−^/^−^ mice

For the ZIKA challenge studies, IFNAR^−^/^−^ mice (*n*=10/group) were immunised once or twice with the ZIKA-prME vaccine or pVax1. The mice were challenge with either 1×10^6^ PFU or 2×10^6^ PFU ZIKV-PR209 virus on day 15 (single immunisation group) or day 21 one week after the second immunisation (two immunisation groups). Post challenge, the animals were weighed and body temperature was measured daily by a subcutaneously located temperature chip. In addition, they were observed for clinical signs of disease twice daily (decreased mobility; hunched posture; hind-limb knuckle walking (partial paralysis), paralysis of one hind limb or both hind limbs) and blood was drawn for viral load determination. The criteria for killing on welfare grounds consisted of 20% weight loss or paralysis in one or both hind limbs.

### Real-time RT-PCR assay for measurement of ZIKV load

The brains from treated mice were immersed in RNAlater (Ambion, Waltham, MA, USA) 4 °C for 1 week, then stored at −80 °C. The brain tissue was then weighed and homogenised in 600 μl RLT buffer in a 2 ml cryovial using a TissueLyser (Qiagen, Valencia, CA, USA) with a stainless steel bead for 6 min at 30 cycles/s. Viral RNA was also isolated from blood with the RNeasy Plus mini kit (Qiagen). A ZIKV specific real-time RT-PCR assay was utilised for the detection of viral RNA from subject animals. RNA was reverse transcribed and amplified using the primers ZIKV 835 and ZIKV 911c and probe ZIKV 860FAM with the TaqMan Fast Virus 1-Step Master Mix (Applied Biosystems, Foster City, CA, USA). A standard curve was generated in parallel for each plate and used for the quantification of viral genome copy numbers. The StepOnePlus Real-Time PCR System (Life Technologies Corporation, Carlsbad, CA, USA) software version 2.3 was used to calculate the cycle threshold (Ct) values, and a Ct value ⩽38 for at least one of the replicates was considered positive, as previously described.^29^ Pre-bleeds were negative in this assay.

### Statistical analysis

Differences in fold increases in antibody titres were compared using Mann–Whitney analysis. Statistical analysis was performed using Graphpad, Prism 4 (Graphpad software, Inc. San Diego, CA, USA). For all the analyses, *P*<0.05 was considered to be significant. Log_10_ transformations were applied to end point binding ELISA titres and whole-virus PRNT_50_ titres.
