# Supplementary figures and images for: In vivo protection against ZIKV infection and pathogenesis through passive antibody transfer and active immunisation with a prMEnv DNA vaccine
Source: NPJ Vaccines. 2016 Nov 10;1:16021–. doi: 10.1038/npjvaccines.2016.21 (PMC5707885; doi:10.1038/npjvaccines.2016.21)

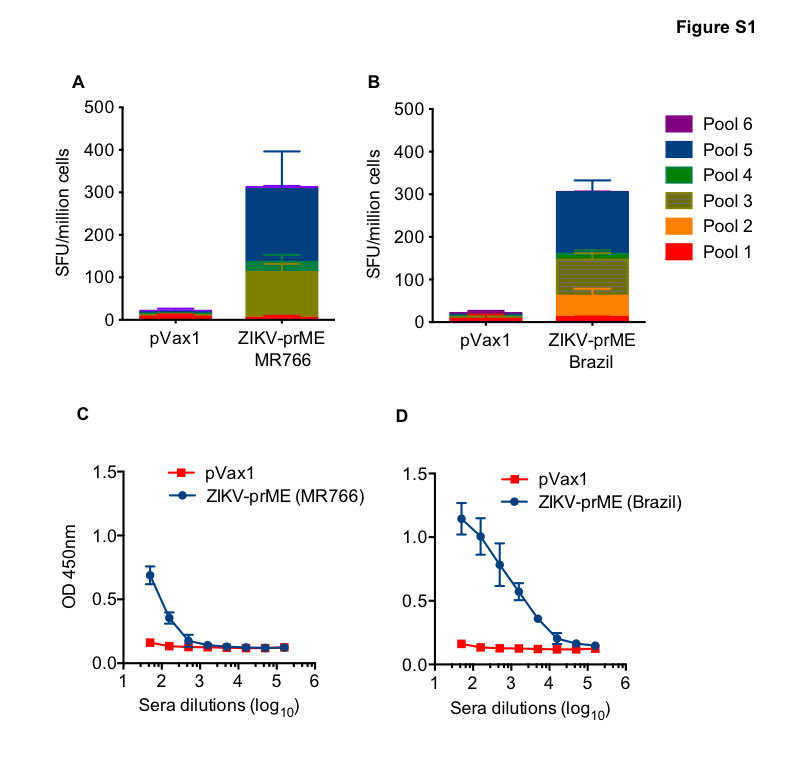

Supplement: Supplementary Figure 1 [file npjvaccines201621-s1.tiff]

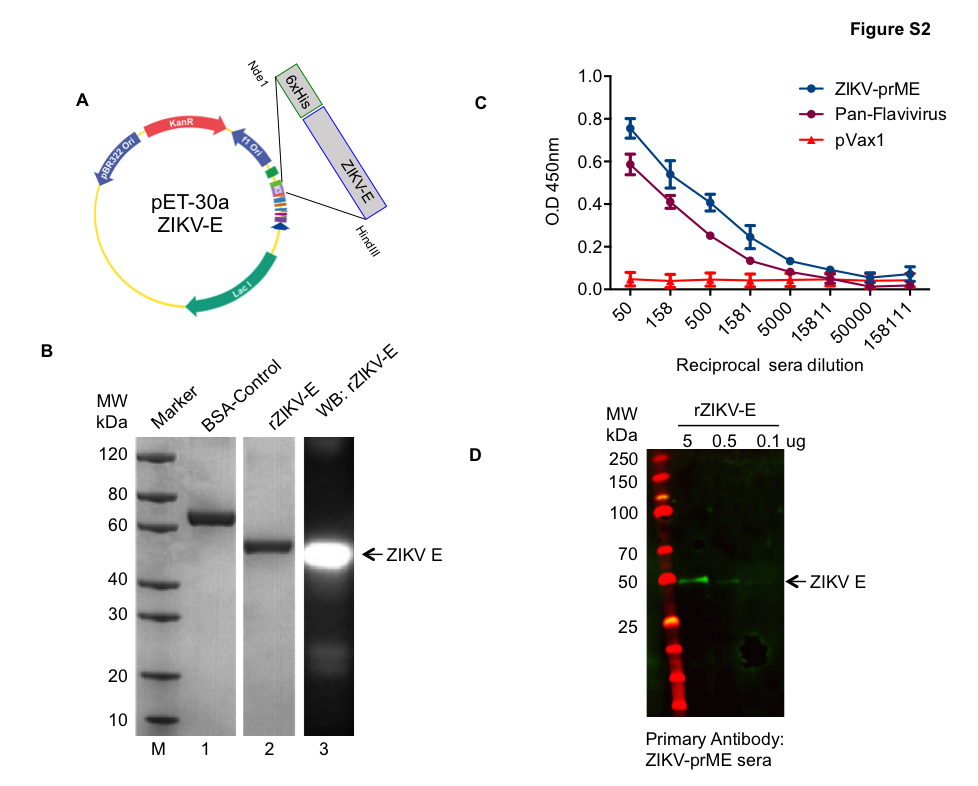

Supplement: Supplementary Figure 2 [file npjvaccines201621-s2.tiff]

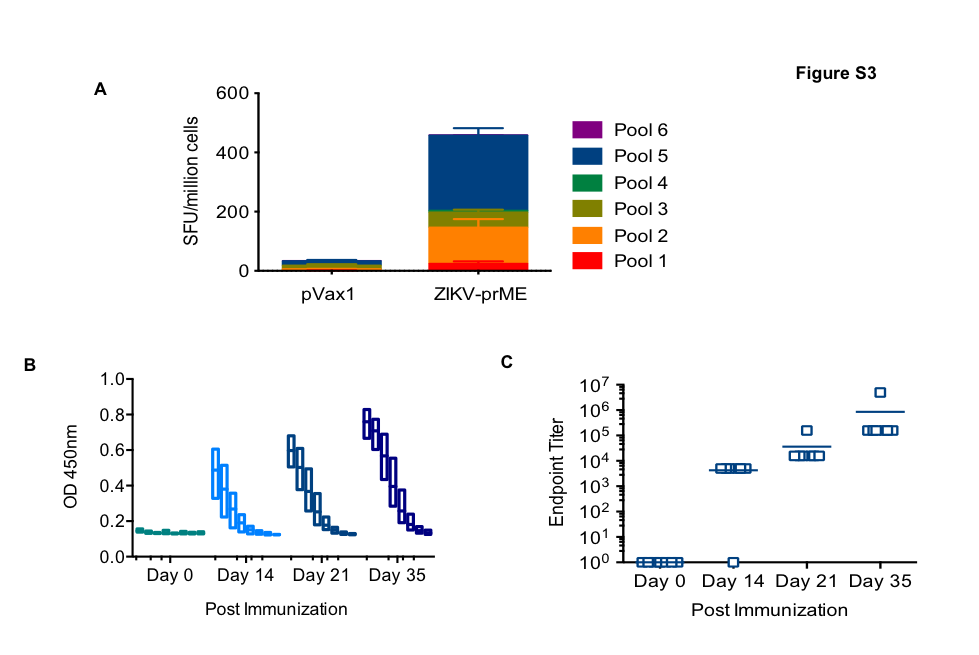

Supplement: Supplementary Figure 3 [file npjvaccines201621-s3.tiff]

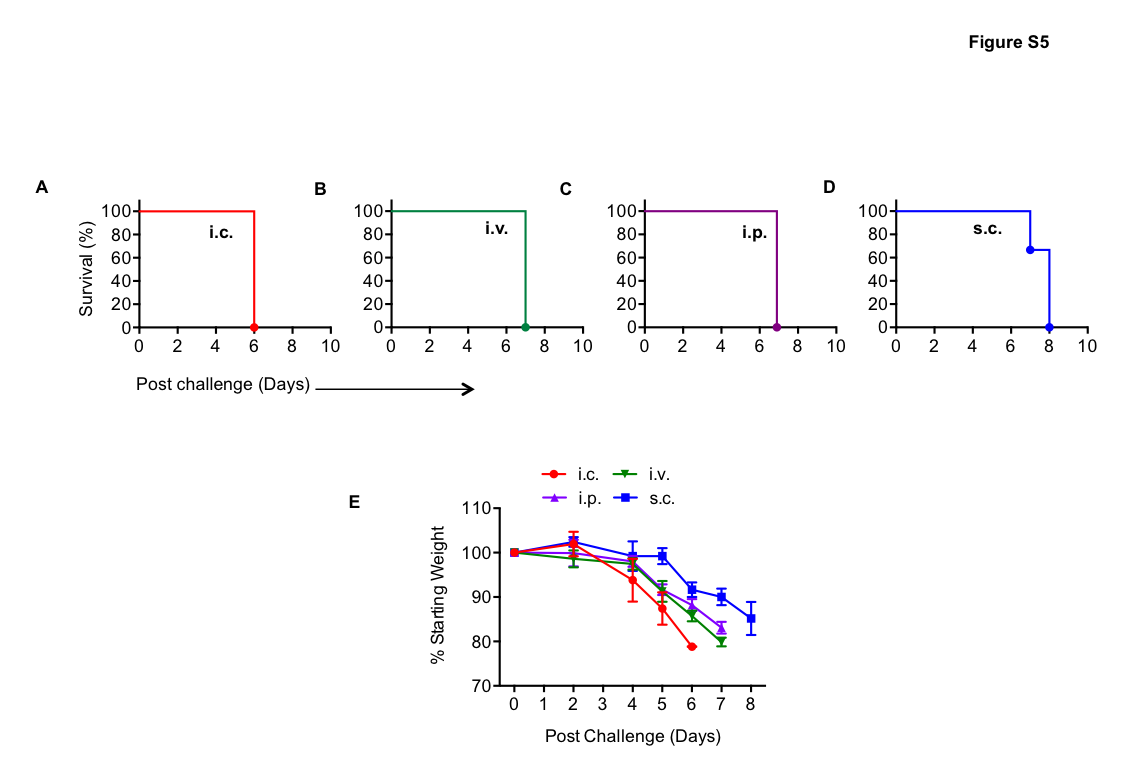

Supplement: Supplementary Figure 5 [file npjvaccines201621-s5.tiff]
