# Supplementary material for: In vivo protection against ZIKV infection and pathogenesis through passive antibody transfer and active immunisation with a prMEnv DNA vaccine
Source: NPJ Vaccines. 2016 Nov 10;1:16021–. doi: 10.1038/npjvaccines.2016.21 (PMC5707885; doi:10.1038/npjvaccines.2016.21)
Supplement: Supplementary Information [file npjvaccines201621-s6.doc]

**SUPPLEMENT FIGURES**

**Figure S1. Characterization of immune responses to ZIKV-prME-MR766 and ZIKV-prME-Brazil vaccine in C57BL/6**

ELISpot and ELISA analysis measuring cellular and antibody responses after vaccination with either ZIKV-prME-MR766 and ZIKV-prME-Brazil DNA vaccines. C57BL/6 mice (n = 4/group) were immunized intramuscularly three times with 25μg of ZIKV-prME-MR766 (A) or ZIKV-prME-Brazil (B) followed by *in vivo*EP. IFN-γ generation, as an indication of cellular immune response induction, was measured by IFN-γ ELISpot. Splenocytes harvested one week after the third immunization were incubated in the presence of one of six peptide pools spanning the entire prM and E proteins. Results are shown in stacked bar graphs. The data represent the average numbers of SFU (spot forming units) per million splenocytes with values representing the mean responses in each ± SEM. (C-D). ELISA analysis measuring binding antibody production in immunized C57BL/6 mice. Binding to rZIKV-E was analyzed for various dilutions of mice sera collected one week post-third immunization (day 35 post- first immunization).

**Figure S2. Expression, purification, and characterization of ZIKV-Envelope protein**

(A) Cloning plasmid for rZIKV-E expression. (B) Characterization of the recombinant ZIKV-E (rZIKV-E) protein by SDS-PAGE and Western blot analysis. Lane 1-BSA control; Lane 2- lysates from pET-28a vector-transformed *E. coli* cultures, purified by nickel metal affinity resin columns and separated by SDS-PAGE after IPTG induction; Lane 3- rZIKV-E purified protein analyzed by Western blot with anti-His antibody; Lane M-protein molecular weight marker. (C) The purified rZIKV-E protein was evaluated for its antigenicity. ELISA plates were coated with rZIKV-E and then incubated with various dilutions of immune sera from the mice immunized with ZIKV-prME vaccine or Pan-flavivirus antibody as positive control. Bound IgG was detected by the addition of peroxidase-conjugated anti-mouse antibody followed by tetramethylbenzidine substrate as described in Materials and Methods. (D) Western blot detection of purified rZIKV-E protein with immune sera from ZIKV-prME immunized mice. Various concentrations of purified rZIKV-E protein were loaded onto an SDS-PAGE gel as described. A dilution of 1:100 immune sera, and goat anti-mouse at 1:15,000 were used for 1 hour at room temperature. After washing, the membranes were imaged on the Odyssey infrared imager. Odyssey protein molecular weight standards were used. The arrows indicate the position of rZIKV-E protein.

**Figure S3. Characterization of immune responses to ZIKA-prME in IFNAR^-^/^-^ mice**

ELISpot and ELISA analysis measuring cellular and antibody responses to ZIKV-prME in IFNAR^-^/^-^ mice. Mice (n = 4/group) were immunized intramuscularly three times at 2-week intervals with 25 μg of ZIKV-prME followed by *in vivo*EP. (A) IFN-γ generation, as an indication of cellular immune response induction, was measured by IFN-γ ELISPOT. (B). ELISA analysis measuring binding antibody production in immunized IFNAR^-^/^-^ mice. Binding to rZIKV-E was analyzed with sera from mice at various time points post immunization. (C). Endpoint titer analysis of anti-ZIKV antibodies produced in immunized IFNAR^-^/^-^ mice.

**Figure S4. Neutralization activity of immune sera from Rhesus Macaques immunized against ZIKV-prME**

Inhibition of infection of Vero (A-B) by ZIKV viruses MR766 and PR209 or SK-N-SH and U87MG cells (C-D) by MR766. Each cell type were mock infected or infected with ZIKV at an MOI of 0.01 PFU/cell in the absence or presence of pooled sera from NHPs immunized with ZIKV-prME vaccine (Wk 6). Zika viral infectivity was analyzed 4 days post infection by indirect immunofluorescence assay (IFA) using sera from ZIKV-prME vaccinated NHPs. Photographs shown of the stained tissue sample slices were taken with a 20x objective (A, C). The bar graph shows the percentage of infected (GFP positive cells) quantified as described in Materials and Methods (B, D).

**Figure S5. ZIKV is virulent to IFNAR^-^/^-^ mice.**

IFNAR^-^/^-^ mice were inoculated via (A) intracranial (i.c); (B) intravenous~~ly~~ (i.v.); (C) intraperitoneial (i.p) and (D) subcutaneous~~ly~~ (s.c.) routes with 10^6^ pfu ZIKV-PR209 virus. Survival analyses of groups are presented as Kaplan-Meier survival curves. These data confirm that ZIKV is virulent in IFNAR^-^/^-^ resulting in morbidity and mortality. (E) Mouse weight change during the course of infection for all the routes.
